# Lettuce as an Effective Remedy in Uremic Pruritus: Review of the Literature Supplemented by an In Silico Study

**DOI:** 10.1155/2022/4231854

**Published:** 2022-03-29

**Authors:** Nazanin Zahra Sepehri, Mohammad Mahdi Parvizi, Sepideh Habibzadeh, Farhad Handjani

**Affiliations:** ^1^Molecular Dermatology Research Center, Shiraz University of Medical Sciences, Shiraz, Iran; ^2^Department of Chemistry, Payame Noor University, Tehran, Iran; ^3^Department of Dermatology, Faculty of Medicine, Shiraz University of Medical Sciences, Shiraz, Iran

## Abstract

Uremic pruritus is a frequent and prominent symptom in patients with advanced or end-stage renal disease. Lack of an effective treatment for kidney disease-associated pruritus often leads to many problems for these patients and makes it difficult to choose an appropriate treatment. The purpose of this evidence-based hypothesis is to share the scientific reasons and related mechanisms in order to claim that lettuce could be useful in the treatment of uremic pruritus. This hypothesis is based on studies related to lettuce and its anti-inflammatory, antioxidant, antidiabetic, sedative, hypnotic, nephroprotective, potassium balancing, and blood purification properties. As a result, we suggest that lettuce could be a good choice for improving and reducing uremic pruritus due to its certain characteristics. Although proof of this hypothesis requires further clinical trial studies, this hypothesis can nevertheless lead to formulating an appropriate therapy for uremic-induced pruritus. By conducting a molecular docking study, we investigated the interactions between nineteen natural bioactive components of lettuce (*Lactuca sativa L.*) and human kappa opioid receptors. The *in silico* docking studies revealed that most of the ligands showed better antipruritic efficacy than gabapentin. Gamma-tocopherol, delta-tocopherol, and campesterol demonstrated the highest binding affinities toward the target protein.

## 1. Introduction

Uremic pruritus, called chronic kidney disease-associated pruritus (CKD-aP), remains as a frequent and prominent symptom in patients with advanced or end-stage renal disease [1]. It affects about one-third of patients on dialysis that is more common with hemodialysis than continuous ambulatory peritoneal dialysis (CAPD). The severity and spatial distribution of pruritus in patients with chronic renal failure may vary considerably over time. The degree of CKD-aP may range from sporadic discomfort to complete restlessness which greatly reduces the patient's quality of life [2, 3].

Uremic pruritus is characterized by daily itching that often tends to worsen at night and may disturb the patient's sleep. The itch may be generalized or localized to one or few areas of the body, most often the back, abdomen, head, and arms [4]. In general, effective treatments for this condition are very limited due to the small number of randomized, placebo-controlled trials, most of which were not conclusive in offering a successful treatment. In addition, the lack of effective treatment is due to an incomplete knowledge of the underlying pathophysiological mechanisms of this condition [5].

The pathogenesis of CKD-aP remains unclear. Studies have shown that parathyroid hormone, histamine, and calcium and magnesium salts were effective in its pathogenesis. Newer hypotheses have focused on opioid receptor irregularities and inflammation as the possible causes of CKD-aP. Accordingly, itching can be very difficult to control because treatment options are limited. The most important treatment approaches are (a) gabapentin, (b) *μ*-opioid and *κ*-opioid receptor antagonists, (c) drugs with anti-inflammatory action, (d) phototherapy, or (e) acupuncture [6–8].

Lettuce (*Lactuca sativa L.*) is one of the most routinely consumed edible crops worldwide. It contains many beneficial constituents, such as polyphenolic compounds, with various biological benefits, including antimicrobial, antioxidant, and anti-inflammatory activities [9].

The purpose of this evidence-based hypothesis is to investigate the scientific reasons and mechanisms to claim that lettuce could be useful as an antioxidant and anti-inflammatory food product in improving and reducing uremic pruritus.

## 2. Materials and Methods

### 2.1. Review of the Literature

The electronic databases, including Scopus, PubMed/MEDLINE, Web of Science, and Google Scholar, were searched for reviewing the literature. In this regard, the keywords of “lettuce,” “*Lactuca sativa L.*,” “Lactuca sativa,” “pruritus,” “itching,” “hypnotic(s),” and “sedative(s)” were used. Time limitation was considered for searching the electronic database. In addition, we reviewed the main sources of traditional Persian medicine (TPM) including “The Canon of Medicine,” “Teb-e-Akbari,” “Eksir-e-Azam” and “Makhzan-al-Advia,” and Al-Shamel fi Sanaat Al-Tebieh,” using Noor software [10], to gather the medicinal use of lettuce claimed by the sages of TPM.

### 2.2. *In Silico* Studies

#### 2.2.1. Protein Preparation

The 3D structure of the target protein, the human kappa opioid receptor, which has been suggested to play an important role in the pathogenesis of CKD-aP [11], was downloaded from the Protein Data Bank (PDB ID: 4DJH) (https://www.rcsb.org) in pdb format. For the preparation of protein structure, water molecules and cocrystal ligands were deleted with the Sequence Editor of AutoDockTools (version 1.5.6); the software was used to add missing hydrogens, and Kollman united atom charges were used to minimize the structure. Finally, the minimized protein structure was converted to .pdbqt format for the docking procedure. The protein active site was determined based on the cocrystal ligand position using the AutoDock 4.2 program.

#### 2.2.2. Ligand Selection and Preparation

Information regarding various constituents of *Lactuca sativa L.*, including minerals, vitamins, and polyphenols, was gathered from the medical literature. These components were screened according to Lipinski's rule of five (molecular weight (MW) not above 500, the number of H-bond acceptors ≤10, the number of H-bond donors ≤5, lipophilicity (log *P*) <5, and molar refractivity (MR) ranging between 40 and 130) [12]. Nineteen bioactive ligands passed the screening step. Gabapentin, which has been reported to be an effective antipruritic agent [13], was selected as a reference ligand.

The chemical structures of the precipitated ligands were retrieved from the PubChem database https://pubchem.ncbi.nlm.nih.gov/) in sdf format and optimized with AM1 energy minimization method using HyperChem 8. The output structures were converted to .pdbqt format using MGLTOOLS 1.5.6 [14]. For the internal validation stage, the cocrystal ligand (JDC) inside the PDB file of the kappa opioid receptor (4DJH) was also extracted and treated the same as other ligands.

#### 2.2.3. Molecular Docking Analysis

Molecular docking is an *in silico* approach that simulates the interactions between proteins and ligands to recognize novel drug candidates. Multiple ligand configurations will generate in this method, and the best pose will be selected based on the minimum docking scores (DS), types of interactions, and root mean square deviation (RMSD) values. The docking protocols were entirely done on validated structures with RMSD values below 2 Å.

The docking simulations were performed using an in-house batch script (DOCKFACE). This application was designed to run AutoDock 4.2 automatically in a stepwise manner. These steps included ligand preparation, receptor preparation, grid maps generation, .dpf files preparation, and finalization of docking runs. The prepared .pdbqt files of the target protein and the ligands were used for docking. The docking grid box was centered on one of the active sites of the protein, *x*=−4.140A°, *y*=−22.228A°,  and *z*=60.007A°, with the box size of 60A° × 70A° ×60A°. To elucidate the collaboration design between 4DJH receptor and the ligands, the Lamarckian genetic algorithm (GA) method was applied. The dockings were carried out on rigid receptor and flexible ligands. For Lamarckian GA, 2,500,000 maximum number of energy evaluations, 27000 maximum generations, 150 population size, a gene mutation rate of 0.02, a crossover rate of 0.8, and 100 number of GA run were applied. All analyses were done on a personal core i7 computer (CPU at 8 MB). The best conformer was selected for each ligand according to the lowest binding energy (−kcal/mol) and better interactions obtained from docking. The interactions formed between the receptor and the ligands were visualized using PLIP (fully automated protein-ligand interaction profiler) [15] and AutoDockTools program (version 1.5.6).

#### 2.2.4. Pharmacokinetic (ADME) Properties Analysis

In order to evaluate favorable drug-like properties of all compounds, pharmacokinetic ADME properties (absorption, distribution, metabolism, and excretion) were evaluated for the bioactive compounds of *Lactuca sativa L.* using SwissADME online tool (https://www.swissadme.ch) [16].

#### 2.2.5. PASS Predictions

In order to search for potential biological activities, the PASS prediction screening program was studied using the PASS online tools (http://www.way2drug.com/passonline/) [17, 18]. The program gives the probabilities on the ratio of Pa (probability to be active) and Pi (probability to be inactive). The probability for biological activity increases when Pa is higher.

## 3. Result and Discussion

### 3.1. Review of the Literature

#### 3.1.1. Anti-Inflammatory and Antioxidant Activity of Lettuce

Antioxidant and anti-inflammatory properties are present in various compounds in vegetables, and the synergic effect of these compounds could increase their therapeutic efficacy. Lettuce has antioxidant and anti-inflammatory properties due to its polyphenol content. In addition, another effective ingredient identified in lettuce is ascorbic acid (AA), which plays an important role in antioxidant processes [19, 20].

Inflammation is a dynamic biological process that occurs as a result of chemical, physical, immune, or biological stimuli. Severe inflammation can lead to damage and destruction of host tissues in various diseases (e.g., type 2 diabetes, cardiovascular diseases, cognitive impairment disorders, brain atrophy, and cancers). In general, overactive macrophages can cause extensive tissue damage. It is widely known that macrophages release inflammatory mediators including cytokines, inducible nitric oxide synthase (iNOS), cyclooxygenase-2 (COX-2), nitric oxide (NO), and reactive oxygen species (ROS) in response to lipopolysaccharides (LPS) in the cell walls of gram-negative bacteria [9, 21].

Evidence has shown that lettuce extract at concentrations of 25–250 g/ml significantly reduces NO production, reduces the expression of iNOS and COX-2, and significantly reduces the release of ROS. Additionally, heme oxygenase-1 (HO-1) can increase cellular antioxidant status by producing antioxidants such as bilirubin that inhibit the expression of iNOS and NO proteins. Pepe et al. showed that lettuce may play an important role in increasing OH-1 expression and reducing the inflammatory response [19].

#### 3.1.2. The Analgesic Properties of Lettuce

Analgesics are a class of medications designed specifically to relieve pain. They include acetaminophen and opioids. Additionally, natural remedies, such as medicinal plants, have been used since ancient times to treat a variety of symptoms, including pain. Accordingly, some studies have shown that compounds in lettuce extract have significant pain-relieving properties [21, 22]. The results of a study by Sayyah et al., which investigated the analgesic effect of lettuce in mice, showed that *Lactuca sativa* extract at a dose of 2 g/kg and 0.5 hours after injection significantly reduced pain and edema. This was due to the presence of triterpenoids, saponins, and simple phenols in the lettuce extract [23].

#### 3.1.3. Antidiabetic Properties of Lettuce

Epidemiological studies have shown that diets containing fruits and vegetables with a particularly high content of polyphenols reduce the risk of chronic diseases, including diabetes, cardiovascular disease, and obesity. Accordingly, anthocyanins, which are considered as polyphenolic pigments, have many antioxidant, anti-inflammatory, and antidiabetic effects [24]. Lettuce is one of the most popular and widely consumed vegetables. In this regard, several reports have suggested that it can improve diabetes by improving glucose metabolism due to its polyphenol content, including anthocyanin [25, 26].

#### 3.1.4. Sedative and Hypnotic Effects of Lettuce

In general, many patients may face numerous side effects and severe drug dependency with the use of long-term hypnotic and sedative medications, including drugs in the benzodiazepine and nonbenzodiazepine families [27]. Hence, many patients tend to use herbal and natural products for alleviating their symptoms [28, 29]. Based on this, a number of chemical agents with sedative and antidepressant effects have been extracted from lettuce. These compounds lead to sedative effects on motor and behavioral activity, improving the muscle-nerve connection, reducing heart rate, and ventricular contraction in the normal heart or during tachycardia. Lactucopicrin and lactosine are among these compounds that have been reported to have sedative activity equal to or greater than ibuprofen [30]. Furthermore, the results of a study by Kim et al showed that lettuce is a rich source for sleep-enhancing substances. Accordingly, pentobarbital in lettuce seeds and leaf extracts could enhance sleep [31]. Moreover, Mosavat et al.'s study showed that lettuce seed syrup was effective for the treatment of insomnia and sleep disturbance in patients with breast cancer [32].

#### 3.1.5. Nephroprotective Effect of Lettuce Extract

Cisplatin (CDDP) is a cytotoxic drug used to treat a variety of cancers. However, an overdose may cause some adverse effects including vomiting, nausea, renal toxicity, and hepatotoxicity [33]. In addition, the accumulation of this compound in the kidneys is very dangerous and could lead to impaired glomerular filtration and to increased serum levels of creatinine and blood urea nitrogen (BUN). Cisplatin also disrupts Na^+^/K^+^ATPase function by reducing the serum levels of potassium and magnesium levels, resulting in cell death [34]. In many Asian countries, sea algae have been used to protect against kidney poisoning caused by cisplatin [33]. In a study, the ethanol extract of lettuce leaf was shown to have a good effect on renal toxicity caused by cisplatin which could improve the renal function parameters, as well as the morphology of cells degraded by cisplatin [35].

#### 3.1.6. Lettuce Potassium Is Suitable for Patients with Kidney Diseases

Reduction of dietary potassium is an important strategy for the management of patients with chronic kidney disease (CKD) who are often receiving drugs that block the renin-angiotensin-aldosterone system (RAAS). Therefore, there is a hypothesis that claims potassium restriction can help patients with chronic kidney disease [36]. Nowadays, there are novel treatment approaches based on a diet containing fruits and vegetables with the right amount of potassium for patients with chronic kidney disease. Accordingly, the results of Sussman et al. study showed that to prevent and manage these conditions, patients undergoing hemodialysis (HD) are recommended to consume a low-potassium diet (2000–3000 mg/day) [25]. Generally, patients with CKD, especially those who suffer from hyperkalemia, should avoid consuming large amounts of raw vegetables, such as lettuce, in order to reduce their potassium intake from meals. However, according to a study by Yoshida et al., low-potassium lettuce, which is found in some Asian countries, contains very low potassium and can be easily consumed by patients with CKD [37].

#### 3.1.7. Use Lettuce to Purify the Blood from Heavy Metals

At least 20 metals have been shown to have toxic effects, and half of them are released into the environment which are hazardous for human health. Because of this, the importance of cleaning the human body from these heavy metals has been stressed [38].

Exposure to cadmium, one of these metals, is very harmful to humans and can lead to fatigue, headache, nausea, vomiting, abdominal cramps, diarrhea, pulmonary emphysema, pulmonary edema, and impaired kidney function. Over the years, many studies have been conducted on plants with higher water content (macrophytes) that can remove toxic metals from the blood. Accordingly, lettuce, as a high biomass product with an extensive root system, can remove heavy metals including cadmium through various mechanisms [39]. As a result, it can be a good food product in order to purify the blood from heavy metals and improve any disease-related effect from these substances.

On the other hand, lettuce could be used as an herbal remedy by having similar effects to opium according to the sources of traditional Persian medicine (TPM). In this setting, the lettuce temperament is cold and wet, so it could be a good choice to reduce body temperature, as well as fever. Furthermore, lettuce has been recommended for blood purification, treatment of jaundice, and pruritus. In addition, sleep induction, analgesia, moistening, diuresis, and laxative effects of lettuce have been mentioned by the sages of TPM. Moreover, the sages have claimed that the narcotic-like effect of lettuce could be stronger than current narcotic medications [40–42].

### 3.2. *In Silico* Study

#### 3.2.1. Molecular Docking Analysis

A molecular docking study was used to evaluate the antipruritic activity of lettuce. The interaction between 19 active compounds of *Lactuca sativa L.* and the human kappa opioid receptor (PDB ID: 4DJH) was investigated. Hydrogen bonds, ion bonds, and *π*-stacking interactions besides hydrophobic and hydrophilic interactions were detected in the docked complexes.

Most of the ligands included in the study interacted with the residues of the active site of the 4DJH receptor by different affinities. The results of the docking score energy (DS), estimated inhibition constant (Ki), amino acid residues involved in hydrogen bonding and hydrophobic interactions, and the number of hydrogen bonds as well as interaction distance between amino acids and the active sites of the compounds are presented in Table 1. The results showed that the best binding affinity for the kappa opioid receptor 4DJH is in the sequence gamma-tocopherol > delta-tocopherol > campesterol > alpha-tocopherol > alpha-lactucerol > riboflavin > thiamine, with lots of convergence points. Other ligands exhibited moderate or poor binding energies, ranging from −2.33 to −6.50 kcal/mol, and caused the formation of several hydrogen bindings with the receptor active site.

**Figure 1:**
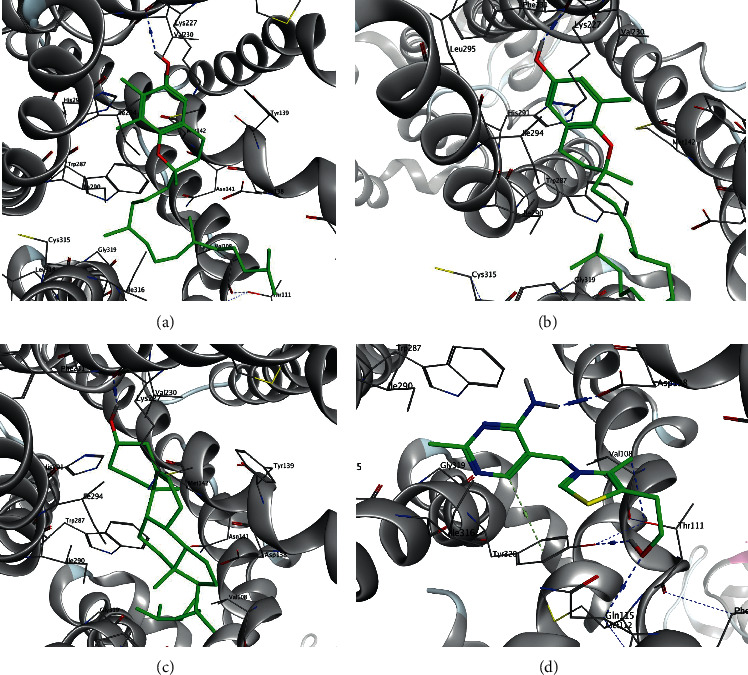
Docking interactions of kappa opioid receptor, 4DJH, with compounds (a) gamma-tocopherol, (b) delta-tocopherol, (c) campesterol, and (d) thiamine. The ligands are represented in green color and hydrogen bonds in blue color.

Gamma-tocopherol and delta-tocopherol showed the best inhibitory potentials with DS = −11.72 kcal/mol and Ki = 2.56 nM and DS = −11.29 kcal/mol and Ki = 5.32 nM, respectively. Visualizing the docked complex interactions revealed that gamma-tocopherol has shared hydrophobic interactions with the residues Val108, Asp138, Val230, Ile290, His291, Ile294, Ile316, and Tyr320 and also has formed a hydrogen bond with Lys227. Delta-tocopherol has shared hydrophobic interactions with Tyr139, lys227, Val230, Trp287, Ile290, Ile294, Tyr312, and Ile316 and has formed two hydrogen bonds with Lys227 and Phe231. Docking simulations of the interactions between some bioactive components of *Lactuca sativa L.* and the 4DJH receptor are presented in Figure 1. The 3D representation of the complexes was performed using Discovery Studio Visualizer 2021.

Comparison of the results for the bioactive components of *Lactuca sativa L.* with the reference drug gabapentin showed that most of the ingredients of this compound have better binding affinities against the kappa opioid receptor, 4DJH, than gabapentin (DS = −5.68 kcal/mol and Ki = 68.98 *μ*M). Table 1 shows that gabapentin interacted with the active site of the target protein through hydrophobic interactions with Val230, Ile290, and Ile294 residues and hydrogen bindings with Asp138. Interestingly, these are the residues involved in the interactions with different components of *Lactuca sativa L.*, such as gamma-tocopherol, delta-tocopherol, campesterol, alpha-tocopherol, and thiamine.

**Figure 2:**
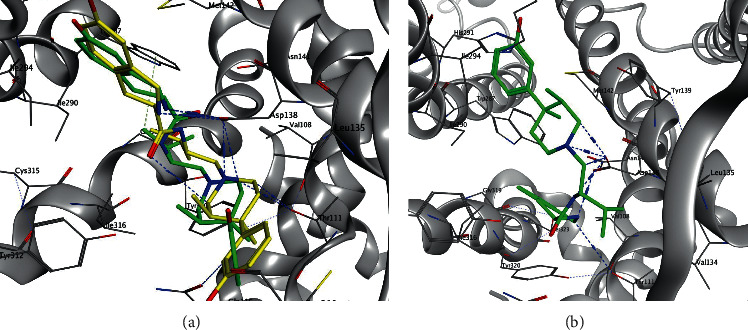
(a) Comparison of two bound conformations of JDC in the kappa opioid receptor's active site: the yellow model shows the crystal orientation, and the redocked result is shown as a green model (b). The key residues surround the structure of JDC in the active site of the kappa opioid receptor.

In the validity assessment step of the docking course, JDC, a natural attached ligand of the kappa opioid receptor (4DJH) with a high affinity for this enzyme [43], redocked to the receptor. As depicted in Table 1, the docking score of −10.18 kcal/mol is obtained for the cocrystal ligand (JDC). Campesterol, gamma-, delta-, and alpha-tocopherol ligands showed more negative binding energies than JDC. The docked conformer of kappa opioid-JDC is demonstrated in Figure 2. It displayed that the best-docked pose of the JDC (green) within the enzyme cavity was consistent with that in the crystal structure (yellow), which points toward the reliability of the molecular docking procedure. The RMSD of docking for JDC compared to its coordination in the crystal structure was 1.35.

Based on RMSD analyses, it can be concluded that the docked protein-ligand complexes were stable, and the docking results could be validated. According to this docking study, the bioactive components of *Lactuca sativa L.* exhibited excellent binding affinity to the 4DJH target protein and could behave like an antagonist at the active site of this kappa opioid receptor.

#### 3.2.2. Pharmacokinetic Properties Analysis

The ADME features of the bioactive components of *Lactuca sativa L.* were evaluated and have been summarized in Table 2. Most of the ligands were fitted with the desired ranges for molecular weight (MW), number of H-bond acceptors, number of H-bond donors, total polar surface area (TPSA), molar refractivity (MR), and parameter for lipophilicity (log *P*). From the water solubility (log *S*) value, we can see that some compounds have good aqueous solubility and some have poor solubility.

#### 3.2.3. PASS Analysis: Biological Activity Predictions

One of the first steps to evaluate whether the compounds could bind to the human kappa opioid receptor was to drive a QSAR study through the PASS prediction screening program.  Table 3 shows the results, and it is predicted that most of the ligands have kappa opioid receptor inhibition activity. To investigate the potential biological activities of gabapentin and components of *Lactuca sativa L.*, those with appreciable binding affinities toward the kappa opioid receptor were selected for further analysis. The nominated compounds were studied using the PASS server. These ligands have revealed predictions for anti-inflammatory, antiallergic, and antipruritic potentials, with Pa ranging from 0.025 to 0.814 (Table 4). Pa > 0.7 shows high pharmaceutical activities, 0.5 < Pa < 0.7 suggests moderate drug activities, and Pa < 0.5 shows poor therapeutic potentials [44]. The values are Pa > Pi, which points to the fact that if the difference between the two values is high, the probability for biological activity increases. The reference drug, gabapentin, showed anti-inflammatory, antipruritic, and kappa opioid receptor inhibitory potentials, validating the predicted results.

## 4. Conclusion

Overall, the lack of an effective treatment for kidney disease-associated pruritus often leads to many problems for these patients. This issue may be because of incomplete knowledge of the underlying pathophysiological mechanisms for pruritus in these patients. This evidence-based hypothesis study claims that lettuce can be a good choice for improving and reducing uremic pruritus in these patients due to its anti-inflammatory, antioxidant, antidiabetic, sedative, hypnotic, nephroprotective, potassium balancing, and blood purification properties. Molecular docking simulation as an *in silico* approach was used to analyze the interaction between natural components of lettuce (*Lactuca sativa L.*) and kappa opioid receptor to determine the effectiveness of this natural herb in the treatment of CKD-aP. This study showed that most of the components of lettuce have a considerably high binding affinity compared to gabapentin as an antipruritic drug. Gamma-tocopherol, delta-tocopherol, campesterol, alpha-tocopherol, alpha-lactucerol, riboflavin, and thiamine had the best docking scores. However, further studies, especially well-designed controlled randomized clinical trials and cohort studies, are recommended to prove this hypothesis in the clinical setting.

## Figures and Tables

**Table 1:** Docking results of the active components of *Lactuca sativa L.* with 4DJH receptor.

Compound	Docking score (kcal/mol)	Number of H-bonds	Estimated inhibition constant, Ki (*μ*M)	Amino acid residues forming H-bond with their length in Å	Hydrophobic interactions
Alanine	−3.24	3	4220	Asp138 (A) H-donor (2.57) Asp138 (A) H-donor (2.69) Asp138 (A) H-donor (2.88)	—
Alpha-lactucerol	−9.66	2	0.083	Thr111 (A) H-donor (3.21, 2.84)	Gln115, Val118, Trp124, Asp138, Ile316
Alpha-linolenic acid	−6.50	1	17.32	Thr111 (A) H-acceptor (2.68)	Val230, Phe231, Ile290, Ile294
Alpha-tocopherol	−10.84	1	0.011	Lys227 (A) H-donor (2.63)	Gln115, Val118, Trp124, Asp138, Tyr139, Lys227, Val230, Ile290, His291, Ile294, Ile316
Arginine	−5.20	6	154.21	Met142 (A) H-donor (3.81) Asp138 (A) H-donor (2.85, 2.66, 2.72) Lys227 (A) H-donor (3.02) His291 (A) H-donor (2.77)	Ile290, Ile294
Ascorbic acid	−4.61	4	416.13	Asp138 (A) H-donor (2.86) Asp138 (A) H-donor (2.74) Ile316 (A) H-donor (2.77) Ile316 (A) H-donor (3.01)	—
Aspartic acid	−2.33	4	19480	Asp138 (A) H-donor (2.72) Asp138 (A) H-donor (2.68) Asp138 (A) H-donor (2.83) Asp138 (A) H-donor (2.96)	—
Betaine	−2.77	1	9260	Ile316 (A) H-donor (2.87)	—
Caffeic acid	−5.33	4	123.69	His291 (A) H-donor (2.79) Asp138 (A) H-donor (2.81) Asp138 (A) H-donor (2.88) Asp138 (A) H-donor (3.39)	Val230, Ile290, Ile294
Campesterol	−11.24	1	0.005	Lys227 (A) H-donor (2.58)	Gln115, Asp138, Tyr139, Trp287, Ile290, Ile294, Ile316, Tyr320
Choline	−3.29	1	3900	Ile316 (A) H-donor (2.78)	
Delta-tocopherol	−11.29	2	0.005	Lys227 (A) H-donor (2.88) Phe231 (A) H-donor (3.91)	Tyr139, lys227, Val230, Trp287, Ile290, Ile294, Tyr312, Ile316
Gamma-tocopherol	−11.72	1	0.002	Lys227 (A) H-donor (3.24)	Val108, Asp138, Val230, Ile290, His291, Ile294, Ile316, Tyr320
Glutamic acid	−3.27	4	4040	Asp138 (A) H-donor (2.63, 2.70, 2.90) His291 (A) H-donor (2.83)	Ile290, Ile294
Niacin	−3.76	1	1740	His291 (A) H-donor (2.72)	Ile294
Pantothenic acid	−3.99	4	1200	Asp138 (A) H-donor (2.77, 2.89, 2.77) Ile316 (A) H-donor (2.77)	Val108, Trp287, Ile290, Ile294, Tyr320
Pyridoxine	−5.02	4	210.20	Asp138 (A) H-donor (2.75, 2.62) Ile316 (A) H-donor (2.84) Thr111 (A) H-donor (3.96)	Val108
Riboflavin	−7.93	3	1.54	Tyr312 (A) H-donor (2.77, 3.01) Gln115 (A) H-acceptor (3.13)	Trp287, Ile316, Tyr320
Thiamine	−7.38	2	3.90	Tyr320 (A) H-donor (2.87) Asp138 (A) H-donor (2.79)	Val108, Thr111, Asp138, Ile290
Gabapentin	−5.68	3	68.98	Asp138 (A) H-donor (3.01, 2.54, 2.70)	Val230, Ile290, Ile294
Cocrystal ligand (JDC)	−10.18	1	0.017	Asp138 (A) H-donor (2.71)	Val108, Gln115, Trp124, Asp138, Trp287, Ile290, Ile294, Ile316

**Table 2:** Pharmacokinetic properties of the bioactive compounds of *Lactuca sativa L.*

Compound	MW (g/mol)	HBA	HBD	TPSA (*A*^°2^)	MR	Log *P*	Log *S*	GI absorption
Alanine	89.09	3	2	63.32	21.01	0.34	1.54	High
Alpha-lactucerol	426.72	1	1	20.23	135.14	4.67	−8.24	Low
Alpha-linolenic acid	278.43	2	1	37.30	88.99	3.36	−4.78	High
Alpha-tocopherol	430.71	2	1	29.46	139.27	6.04	−8.6	Low
Arginine	174.20	4	4	127.72	44.54	0.27	2.05	Low
Ascorbic acid	176.12	6	4	107.22	35.12	−0.31	0.23	High
Aspartic acid	133.10	5	3	100.62	27.59	−0.09	1.98	High
Betaine	117.15	2	0	40.13	28.35	−2.19	−0.35	Low
Caffeic acid	180.16	4	3	77.76	47.16	0.97	−1.89	High
Campesterol	400.68	1	1	20.23	128.42	4.97	−7.54	Low
Choline	104.17	1	1	20.23	29.69	−2.14	−0.1	Low
Delta-tocopherol	402.65	2	1	29.46	129.34	5.44	−7.98	Low
Gamma-tocopherol	416.68	2	1	29.46	134.31	5.16	−8.29	Low
Glutamic acid	147.13	5	3	100.62	32.40	0.40	1.84	High
Niacin	123.11	3	1	50.19	31.20	0.86	−1.26	High
Pantothenic acid	219.23	5	4	106.86	52.21	0.95	−0.06	High
Pyridoxine	169.18	4	3	73.58	43.48	0.80	−0.64	High
Riboflavin	376.36	8	5	161.56	96.99	1.63	−1.31	Low
Thiamine	265.35	3	2	104.15	73.26	−1.60	−2.32	High

MW, molecular weight; HBA, number of H-bond acceptor; HBD, number of H-bond donor; TPSA, total polar surface area; MR, molar refractivity; log *P*, lipophilicity; log *S*, water solubility.

**Table 3:** QSAR observation results obtained from the PASS Server for lettuce (*Lactuca sativa L.*) components.

Compound	Kappa opioid receptor antagonist
Alanine	Yes
Alpha-lactucerol	Yes
Alpha-linolenic acid	Yes
Alpha-tocopherol	No
Arginine	Yes
Ascorbic acid	Yes
Aspartic acid	Yes
Betaine	Yes
Caffeic acid	Yes
Campesterol	No
Choline	(Result not found)
Delta-tocopherol	Yes
Gamma-tocopherol	Yes
Glutamic acid	Yes
Niacin	Yes
Pantothenic acid	Yes
Pyridoxine	Yes
Riboflavin	No
Thiamine	(Result not found)
Gabapentin	Yes

**Table 4:** PASS prediction of the standard drug and the nominated bioactive compounds of *Lactuca sativa L.*

Compound	Pa	Pi	Activity
Alpha-lactucerol	0.749	0.010	Anti-inflammatory
	0.229	0.228	Anti-inflammatory, ophthalmic
	0.250	0.065	Nonsteroidal anti-inflammatory agent
	0.025	0.005	Anti-inflammatory steroid
	0.660	0.010	Antipruritic
	0.512	0.035	Antipruritic, allergic
	0.412	0.012	Antipruritic, nonallergic
	0.283	0.103	Opioid kappa 3 receptor antagonist

Alpha-tocopherol	0.814	0.006	Anti-inflammatory
	0.587	0.003	Anti-inflammatory, ophthalmic
	0.203	0.098	Nonsteroidal anti-inflammatory agent
	0.599	0.016	Antipruritic
	0.252	0.202	Antipruritic, allergic
	0.217	0.079	Antipruritic, nonallergic
	0.328	0.080	Antiallergic

Campesterol	0.502	0.056	Anti-inflammatory
	0.457	0.005	Anti-inflammatory, ophthalmic
	0.043	0.003	Anti-inflammatory steroid
	0.774	0.004	Antipruritic
	0.679	0.004	Antipruritic, allergic
	0.502	0.003	Antipruritic, nonallergic

Delta-tocopherol	0.720	0.013	Anti-inflammatory
	0.436	0.006	Anti-inflammatory, ophthalmic
	0.351	0.076	Antipruritic
	0.349	0.106	Antipruritic, allergic
	0.222	0.076	Antipruritic, nonallergic
	0.251	0.151	Opioid kappa 3 receptor antagonist

Gamma-tocopherol	0.775	0.008	Anti-inflammatory
	0.562	0.003	Anti-inflammatory, ophthalmic
	0.222	0.083	Nonsteroidal anti-inflammatory agent
	0.222	0.155	Anti-inflammatory, intestinal
	0.511	0.033	Antipruritic
	0.338	0.113	Antipruritic, allergic
	0.238	0.066	Antipruritic, nonallergic
	0.220	0.164	Antiallergic
	0.223	0.205	Opioid kappa 3 receptor antagonist

Gabapentin	0.320	0.144	Anti-inflammatory
	0.370	0.032	Anti-inflammatory, intestinal
	0.357	0.024	Anti-inflammatory, ophthalmic
	0.298	0.043	Nonsteroidal anti-inflammatory agent
	0.307	0.109	Antipruritic
	0.422	0.072	Antipruritic, allergic
	0.283	0.043	Antipruritic, nonallergic
	0.708	0.009	Analgesic
	0.317	0.027	Analgesic stimulant
	0.357	0.040	Opioid kappa 3 receptor antagonist

Cocrystal ligand (JDC)	0.301	0.115	Antipruritic
	0.306	0.078	Opioid kappa 3 receptor antagonist

## Data Availability

The data will be available on request through email to the corresponding author.
